# Tubulointerstitial nephritis in primary Sjögren syndrome: clinical manifestations and response to treatment

**DOI:** 10.1186/s12891-015-0858-x

**Published:** 2016-01-05

**Authors:** Rhys D. R. Evans, Christopher M. Laing, Coziana Ciurtin, Stephen B. Walsh

**Affiliations:** UCL Centre for Nephrology, UCL Medical School, Rowland Hill Street, London, NW3 2PF UK; Department of Rheumatology, University College London Hospital, NHS Trust, 3rd Floor Central, 250 Euston Road, London, NW1 2PG UK

**Keywords:** Epithelial inflammation, Glomerulonephritis, Immunosuppression, Renal tubular acidosis, Sjögren syndrome, Tubulointerstitial nephritis

## Abstract

**Background:**

Primary Sjögren syndrome (pSS) is a common autoimmune condition which primarily affects epithelial tissue, often including the kidney causing either tubulointerstitial nephritis (TIN) or more rarely, an immune complex related glomerulonephritis.

**Methods:**

We describe the clinical, biochemical and histological characteristics of 12 patients with pSS related TIN and their response to treatment with antiproliferative agents. All 12 patients were investigated and treated at the UCL Centre for Nephrology in London.

**Results:**

All patients had TIN demonstrated via needle biopsy; immunophenotyping showed that the interstitial infiltrate was predominantly a CD4+ T-cell infiltrate. Urinary acidification testing demonstrated distal renal tubular acidosis in 8 patients. Proximal tubular dysfunction was present in 5 patients. All but 1 patient were treated with antiproliferative agents and most also with a reducing course of steroids. In the treated patients, there was a significant improvement in the serum creatinine and measured GFR.

**Conclusion:**

Patients with pSS TIN have significant renal impairment and other functional tubular defects. There is a mononuclear lymphocytic infiltrate on renal biopsy and this appears to be mainly a CD4+ T-cell infiltrate. Treatment with mycophenolate (and corticosteroids) improves the renal function in patients with pSS TIN.

## Background

The Swedish ophthalmologist Henrik Sjögren described a disease characterized by oral and conjunctival dryness (the ‘sicca syndrome’) in 1933 [1]. This disease, ‘Sjögren syndrome’, may occur in isolation (primary Sjögren syndrome or pSS) or secondary to other autoimmune diseases. The epithelial inflammation that leads to failure of lacrimal and salivary secretion can also lead to the destruction of other epithelial tissues such as airway, biliary, pancreatic and renal epithelia [2].

Renal disease in pSS is common; its prevalence in some series of pSS being as high as 42 % [3].

Rarely, pSS can cause a glomerular lesion with decreased excretory renal function and proteinuria. The lesion itself is typically a membranoproliferative glomerulonephritis (MPGN), but can present with a number of other glomerular lesions (e.g. membranous nephropathy). This is due to immune complex deposition associated with B-cell expansion, cryoglobulinaemia and lymphoma [4, 5].

However, epithelial inflammation in pSS typically causes tubulointerstitial nephritis, the commonest renal lesion in pSS [6]. Although this may cause renal impairment, it also causes renal tubular lesions which may be more difficult to diagnose.

Here, we describe 12 patients with a tubulointerstitial nephritis secondary to pSS, their demographic, biochemical, immunological and histological characteristics along with their response to immunosuppression.

## Methods

Patients were referred to the UCL Centre for Nephrology tubular clinic and all underwent investigation with serum biochemistry, immunology, and renal biopsy. Urinary acidification testing was performed in 9 patients. GFR was estimated in all patients using the Modification of Renal Diet (MDRD eGFR) equation. 6 patients underwent 3-dose ^51^Chromium EDTA-GFR (^51^Cr-GFR) measurements before and after treatment. All patients with pSS who underwent renal biopsy from January 2007-December 2014 were included in this analysis.

The diagnosis of pSS was based on the 2002 American European consensus criteria [7], all patients satisfied the same criteria; ocular and oral symptoms, positive Schirmer test and positive Ro/La antibody status.

All patients who were suspected of having distal renal tubular acidosis (dRTA) underwent urinary acidification testing with a furosemide and fludrocortisone test [8] or an ammonium chloride test [9]. Briefly, the patient is administered either 40 mg of furosemide and 1 mg of fludrocortisone, or 0.1 mg/kg of ammonium chloride orally and the urine pH is monitored hourly. A fall in the urine pH to less than 5.3 represents normal urinary acidification; a failure to do so is diagnostic of dRTA.

Renal biopsy tissue was uniformly fixed in paraffin and sections were stained with hematoxylin and eosin. Immunophenotyping was performed by additional immunostaining with polyclonal antibodies to CD3, CD4, CD8, CD20, CD1a and CD138.

Where slides were available to review, we scored each biopsy to assess the nature of the inflammatory cell infiltrate and the degree of interstitial scarring. The infiltrate was scored as being either patchy or diffuse, the approximate amount of interstitium involved (<25 %, 25–50 %, 50–75 %, >75 %); the intensity of the infiltrate was scored as 1+ (light), 2+ (moderate) or 3+ (heavy); the predominant cell type in the infiltrate was recorded as was the amount of scarring, again recorded as 1–3 +.

Patients were treated with an antiproliferative agent, mycophenolate mofetil (MMF) or, in one case, azathioprine and where possible, a short reducing course of corticosteroid. The dose of MMF was titrated according to symptoms and recovery of renal function, and, in hypergammaglobulinemic patients, with the aim of bringing the IgG level within the normal range.

Statistical analysis was performed using GraphPad Prism 5.03, statistical significance was determined using the student’s *t*-test for Gaussian and Wilcoxon matched pair signed rank test for non-Gaussian distributed data.

### Ethical considerations

All patients provided written informed consent for participation in the study and ethical approval was attained from the local ethics board (Great Ormond Street Hospital Ethics Committee). The authors have respected participants’ rights to privacy by de-identifying all patients data reported. By doing so, the authors feel they have maintained anonymity and confidentiality in accordance with local data protection laws.

### Case vignettes

Case 1: A 54-year-old woman with pSS was referred with impaired renal function (eGFR 28 ml/min/1.73 m^2^). She complained of sicca symptoms, fatigue, urinary frequency and nocturia. She had raised inflammatory markers, a positive ANA, anti-Ro antibody and rheumatoid factor. A renal biopsy demonstrated TIN and she was commenced on MMF 2 g/day and a reducing course of prednisolone 30 mg. Her renal function remained stable for 5 years but the patient unilaterally stopped her immunosuppression; and her serum creatinine rose from 168 to 230 μmol/L. A second biopsy demonstrated TIN with significant scarring and mild glomerular changes (mesangial matrix expansion). Immunosuppression was restarted with renal function settling to baseline.

Case 2: A 52-year-old woman became unwell after a holiday in Pakistan. She presented with severe weakness, hypokalaemia and metabolic acidosis (serum bicarbonate 12 mmol/L). She was referred for investigation of dRTA, which was confirmed on urinary acidification testing. On direct questioning she also reported sicca symptoms, fatigue and arthralgia. She was ANA, anti-Ro and anti-La antibody positive. She was treated with potassium citrate and hydroxychloroquine 400 mg/day. She had deteriorating renal function (serum creatinine rose from 88 to 124 μmol/L) and her renal biopsy demonstrated TIN. MMF (500 mg/day) was started resulting in improvement in her renal function but not in her symptoms.

Case 3: A 36-year-old woman with known pSS was referred with persistent hypokalemic acidosis after elective cholecystectomy. She had dRTA confirmed on urinary acidification testing. There was also proximal tubular dysfunction (phosphate wasting and low molecular weight (LMW) proteinuria) and suggestion of a concentrating defect (low early morning urinary osmolality). Renal biopsy demonstrated TIN and she was treated with bicarbonate and potassium supplements in addition to low dose prednisolone (5 mg/day), hydroxychloroquine (400 mg/day) and MMF (1 g/day). Higher doses of MMF were prohibited by gastrointestinal side effects. She had progressive renal dysfunction (serum creatinine rose from 79 to 115 μmol/L) and a repeat biopsy 4 years after the first demonstrated ongoing TIN with worse scarring. Treatment with rituximab was attempted but abandoned after she developed an anaphylactoid reaction. Therefore MMF was titrated to 2 g/day and the prednisolone dose doubled. Despite this, she has slowly progressive deterioration in her GFR and severe extra-renal features (sicca symptoms, parotitis and arthritis).

Case 4: A 45-year-old woman with known pSS was referred with impaired renal function (eGFR 40 ml/min). She had defective urinary acidification on testing. She was treated with bicarbonate supplements. A renal biopsy confirmed TIN. Her serum creatinine deteriorated from 124 to 159 μmol/L and azathioprine 50 mg/day was started. Her renal function stabilized but her sicca symptoms remain.

Case 5: A 71-year-old woman had longstanding sicca symptoms and chronic hypokalaemia. She presented to hospital with diarrhea with severe hypokalaemia (2.1 mmol/L) and paralysis. The diarrhea was due to a colonic villous adenoma, for which she underwent a hemicolectomy. She was referred to our service for her hypokalaemia which persisted after her surgery. She reported sicca symptoms, arthralgia and systemic upset with associated positive pSS serology. dRTA was confirmed by urinary acidification testing. She underwent renal biopsy which demonstrated TIN with heavy staining for C9 along tubular basement membranes (negative staining for immunoglobulin). Potassium and bicarbonate supplements were commenced along with prednisolone 20 mg/day and hydroxychloroquine 200 mg/day. She was intolerant of both of these (dyspepsia and rash respectively) and developed worsening renal function off immunosuppression (serum creatinine rose from 97 to 159 μmol/L). MMF (1 g/day) was introduced with resolution of her renal impairment and improvement in her extra-renal symptoms.

Case 6: A 54-year-old woman was referred having passed a pure calcium phosphate stone, suggestive of dRTA. She had renal impairment (eGFR 32 ml/min/1.73 m^2^), imaging which demonstrated multiple stones bilaterally, and a long history of sicca symptoms with systemic upset. Her pSS serology was positive, she had dRTA confirmed on urinary acidification testing and had evidence of proximal tubular dysfunction (LMW proteinuria). A renal biopsy demonstrated TIN. She was treated with potassium citrate, a reducing course of prednisolone, MMF 1 g/day, and hydroxychloroquine 200 mg/day. Consequently, her renal function and symptoms improved.

Case 7: A 48-year-old woman presented to her GP with tiredness. She was found to have renal impairment (eGFR 39 ml/min/1.73 m^2^) and nephrocalcinosis. On direct questioning, she reported sicca symptoms and a rash. Her pSS serology was positive and she underwent renal biopsy, which demonstrated TIN with areas of calcification. She also had an incidental finding of thin glomerular basement membranes on electron microscopy. She had a systemic acidosis with an inappropriately high urinary pH, confirming dRTA. She was treated with potassium and bicarbonate supplementation, low dose steroid and MMF 500 mg/day; her renal function and symptoms improved.

Case 8: A 43-year-old woman was referred to our center with renal impairment (eGFR 36 ml/min/1.73 m^2^) and nephrocalcinosis. She reported longstanding sicca symptoms with systemic upset. She was acidotic (serum bicarbonate 18 mmol/L) and hypokalemic (2.8 mmol/L), and had dRTA confirmed by urinary acidification testing. She also had proximal tubular dysfunction (phosphate wasting and LMW proteinuria) and serology for pSS was positive. Renal biopsy demonstrated TIN with positive staining for C9 along tubular basement membranes. She was treated with potassium citrate supplements, a reducing course of prednisolone, and MMF 1.5 g/day. Her renal function stabilized, her electrolytes normalized, and her extra renal symptoms substantially improved.

Case 9: A 51-year-old woman was referred with unexplained renal impairment (eGFR 30 ml/min/1.73 m^2^). She had a long history of dry eyes for which she had been using artificial tears. pSS serology was positive and she underwent renal biopsy which demonstrated TIN. Subsequent urinary acidification testing demonstrated dRTA. She was treated with a reducing course of prednisolone and MMF 1.5 g/day. Her renal function improved but her sicca and non-specific symptoms persisted.

Case 10: A 61-year-old woman was referred with renal impairment (eGFR 43 ml/min/1.73 m^2^). She was non-specifically unwell with positive pSS serology and sicca symptoms. She underwent renal biopsy, which demonstrated TIN, and she was treated with a weaning course of prednisolone. Renal function was stable for 4 years off treatment but underwent further renal biopsy to reassess interstitial inflammation. This showed ongoing areas of TIN with increased chronic damage when compared to the initial biopsy. Immunosuppression with both MMF 500 mg/day and subsequently azathioprine 75 mg/day has been complicated by anemia and leucopenia. A bone marrow aspiration and biopsy revealed myelodysplastic changes only. She remains off immunosuppression currently with stable renal function (eGFR 45 ml/min/1.73 m^2^) and limited symptoms.

Case 11: A 72-year-old woman with longstanding systemic upset and arthralgia was initially misdiagnosed with rheumatoid arthritis. She subsequently developed impaired renal function (eGFR 46 ml/min/1.73 m^2^). She had mild sicca symptoms and her pSS serology was positive; a renal biopsy demonstrated TIN with C9 staining along tubular basement membranes. She was treated with MMF 1 g/day. Renal function improved as did her symptoms.

Case 12: A 53-year-old man was admitted with a painful peripheral neuropathy. He complained of sicca symptoms, was found to have renal impairment (eGFR 50 ml/min/1.73 m^2^), and developed a vasculitic rash. Cryoglobulins were present, there was hypocomplementaemia, pSS serology was positive, and a renal biopsy demonstrated TIN with mild mesangial proliferation. He was treated with a reducing course of prednisolone (initially 40 mg/day) and MMF 1 g BD. His renal function improved and his symptoms have settled.

## Results

The series encompasses twelve patients, 11 of these are female, with a median age of 52.5 (interquartile range (IQR) 13.5 years). At presentation the patients tended toward a metabolic acidosis (median serum bicarbonate 21 mmol/L, IQR 5.75), and had moderate renal impairment (median serum creatinine 133 μmol/L, IQR 53.2), but little or no hypokalaemia (median serum potassium 4.2 mmol/L, IQR 1.25).

The diagnosis of pSS was not known in 8 (66 %) patients prior to investigation of their renal impairment. All patients were Ro positive and 61 % were La positive. Rheumatoid factor was raised in 67 % (median 388 IU/L, IQR 630.8), complement factors C3 and C4 were preserved (median 108 and 16.0 mg/dL, IQR 49 and 6, respectively) and there was hypergammglobulinemia at presentation in 70 % (median IgG 19.8 g/L, IQR 10) and the erythrocyte sedimentation rate tended to be high (median 50 mm/h, IQR 72.75) (Table 1).

**Table 1:** Demographic, biochemical and immunological data for the 12 patients with pSS

Patient	Age	Sex	Presentation/reason for referral	Extra-renal clinical features	ANA	Anti-Ro	Anti-La	RF (0–20 IU/ml)	C3 (70–165 mg/dL)	C4 (16–54 mg/dL)	IgG (7-16 g/L)	Serum protein electrophoresis	ESR (mm/hr)	Creatinine (μmol/L)	Urine PCR (mg/mmol)
1	54	Female	Renal impairment Urinary Symptoms	Sicca, Non-specific (generally unwell, fatigue, poor energy)	positive (>1/1000 fine speckled)	Positive	Negative	145	128	15	24.9	Polyclonal increase in immunoglobulins	107	168	0
2	52	Female	Hypokalaemic acidosis with paralysis	Sicca, Non-specific (generally unwell, fatigue, poor energy), arthralgias	positive (>1/1000 fine speckled)	Positive	Positive	N/A	137	29	27.5	Polyclonal increase in immunoglobulins	88	97	27
3	36	Female	Hypokalaemic acidosis	Sicca, Non-specific (generally unwell, fatigue, poor energy), arthralgias, parotitis, low mood.	positive (>1/1000 fine speckled)	Positive	Positive	718	148	21	24.8	Polyclonal increase in immunoglobulins	35	88	102
4	45	Female	Renal impairment	Sicca	positive (>1/1000 fine speckled)	Positive	Positive	743	139	15	37.6	Polyclonal increase in immunoglobulins	121	124	N/A
5	71	Female	Hypokalaemic acidosis with paralysis	Sicca, Non-specific (generally unwell, fatigue, poor energy), arthralgias	positive (>1/1000 fine speckled)	Positive	Negative	618	124	21	21.3	Polyclonal increase in immunoglobulins	86	106	101
6	54	Female	Renal impairment Stones	Sicca, Non-specific (generally unwell, fatigue, poor energy)	positive (>1/1000 fine speckled)	Positive	Positive	69	89	16	18.5	N/A	5	186	50
7	48	Female	Renal impairment Nephrocalcinosis	Sicca, Non-specific (generally unwell, fatigue, poor energy), rash	positive (>1/1000 fine speckled)	Positive	Positive	93	N/A	N/A	14	Polyclonal increase in immunoglobulins	33	133	127
8	43	Female	Renal impairment Nephrocalcinosis	Sicca, Non-specific (generally unwell, fatigue, poor energy)	positive (>1/1000 fine speckled)	Positive	Positive	<20	108	21	19.7	Polyclonal increase in immunoglobulins	N/A	141	46
9	51	Female	Renal impairment	Sicca, Non-specific (generally unwell, fatigue, poor energy)	positive (>1/1000 fine speckled)	Positive	Negative	<20	70	16	19.9	N/A	5	168	0
10	61	Female	Renal impairment	Sicca, non-specific (generally unwell, fatigue, poor energy)	positive (>1/1000 fine speckled)	Positive	Positive	158	85	20	13.7	Polyclonal increase in immunoglobulins	25	115	0
11	72	Female	Renal impairment	Sicca, non-specific (generally unwell, fatigue, poor energy), arthralgia and GI upset.	positive (>1/1000 fine speckled)	Positive	Negative	N/A	88	14	17.6	Paraprotein with immunoparesis	N/A	133	N/A
12	53	Male	Renal impairment	Sicca, neuropathy, vasculitic rash, cryoglobulinaemia	positive (>1/1000 fine speckled)	Positive	Negative	1370	96	3	12.4	Type 1 IgM kappa cryoglobulin	65	133	40

**Fig. 1:**
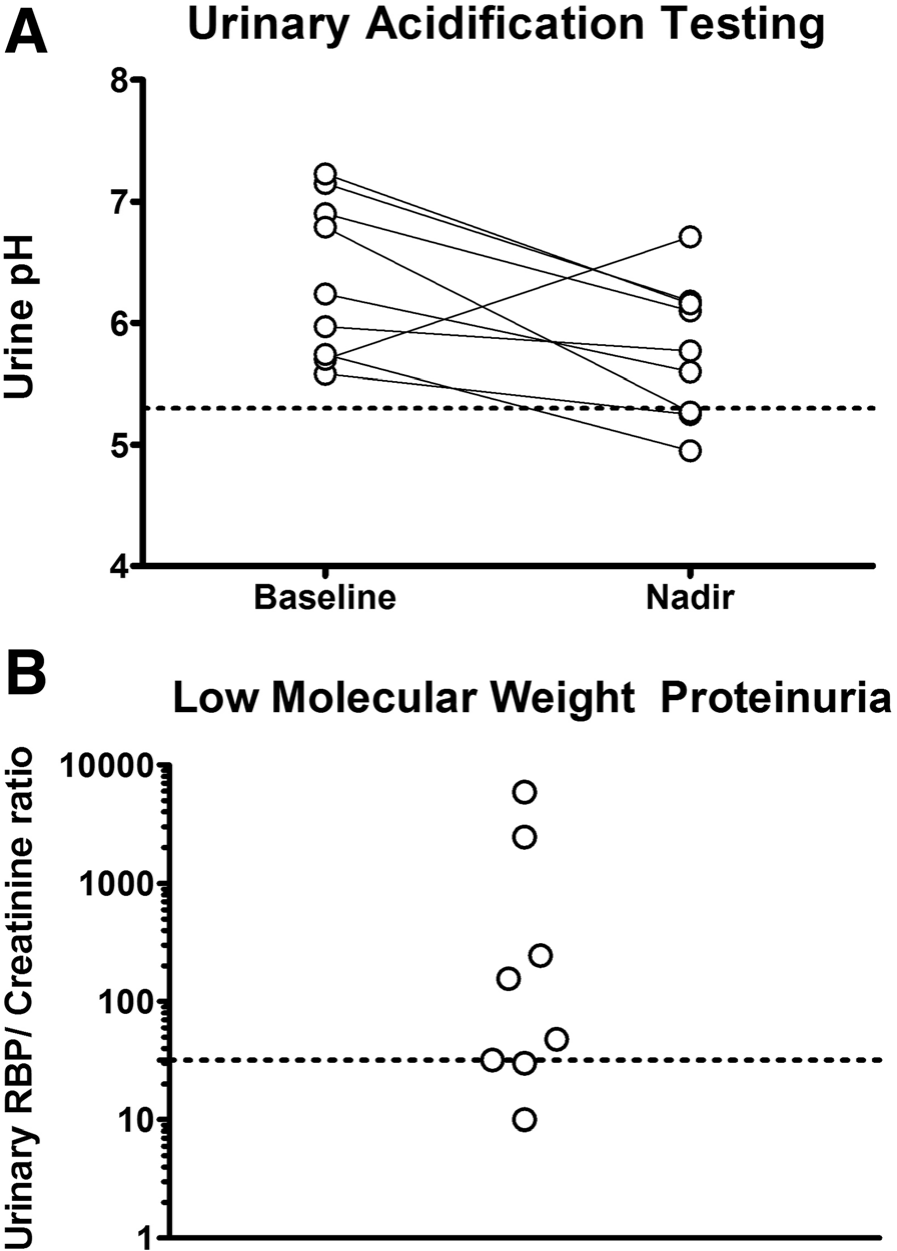
Demonstrates distal and proximal tubular dysfunction. Panel **a** shows urinary acidification tests. Baseline and nadir urine pH values are shown. The dotted line represents the threshold urine pH of 5.3 that determines normal urinary acidification. Panel **b** shows urinary RBP/creatinine ratio. The dotted line shows the upper limit of normal (32 μg/mmol)

9 patients had urinary acidification testing with furosemide and fludrocortisone or ammonium chloride. All but two of nine patients had abnormal urinary acidification, indicating dRTA (median baseline urine pH 6.24, IQR 1.3; median nadir urine pH 5.77, IQR 0.91); of the remaining two, one had an equivocal result (Fig. 1). A further 2 patients had a systemic acidosis and an inappropriately alkaline pH at baseline. Overall, 75 % had evidence of abnormal urinary acidification.

Proximal tubular dysfunction was assessed in 8 patients by measuring urinary retinol binding protein (RBP). The urinary RBP/Creatinine ratio was abnormally high in 5 patients (median 102 μg/mmol, IQR 1867, normal range 4–32 μg/mmol), and 2 patients had extremely high ratios (as high as 5885 μg/mmol).

All patients underwent a renal biopsy. Histology revealed a tubulointerstitial nephritis in all patients. Of the 14 biopsies performed in this series, 11 were available for us to review the tissue in addition to having the formal report.

The inflammatory cell infiltrate was diffuse in two of the biopsies and patchy in the remainder, and the intensity was rated as 1+ (light) in 5, 2+ (moderate) in 5 and 3+ (heavy) in 1. The proportion of interstitium affected by the infiltrate was <25 % in 3, 25–50 % in 2, 50–75 % in 2 and >75 % in 4. In all cases the predominant cell type was mononuclear lymphocytes. Scarring was judged to be 1+ (light) in 6, 2+ (moderate) in 4, and 3+ (heavy) in 1.

**Fig. 2:**
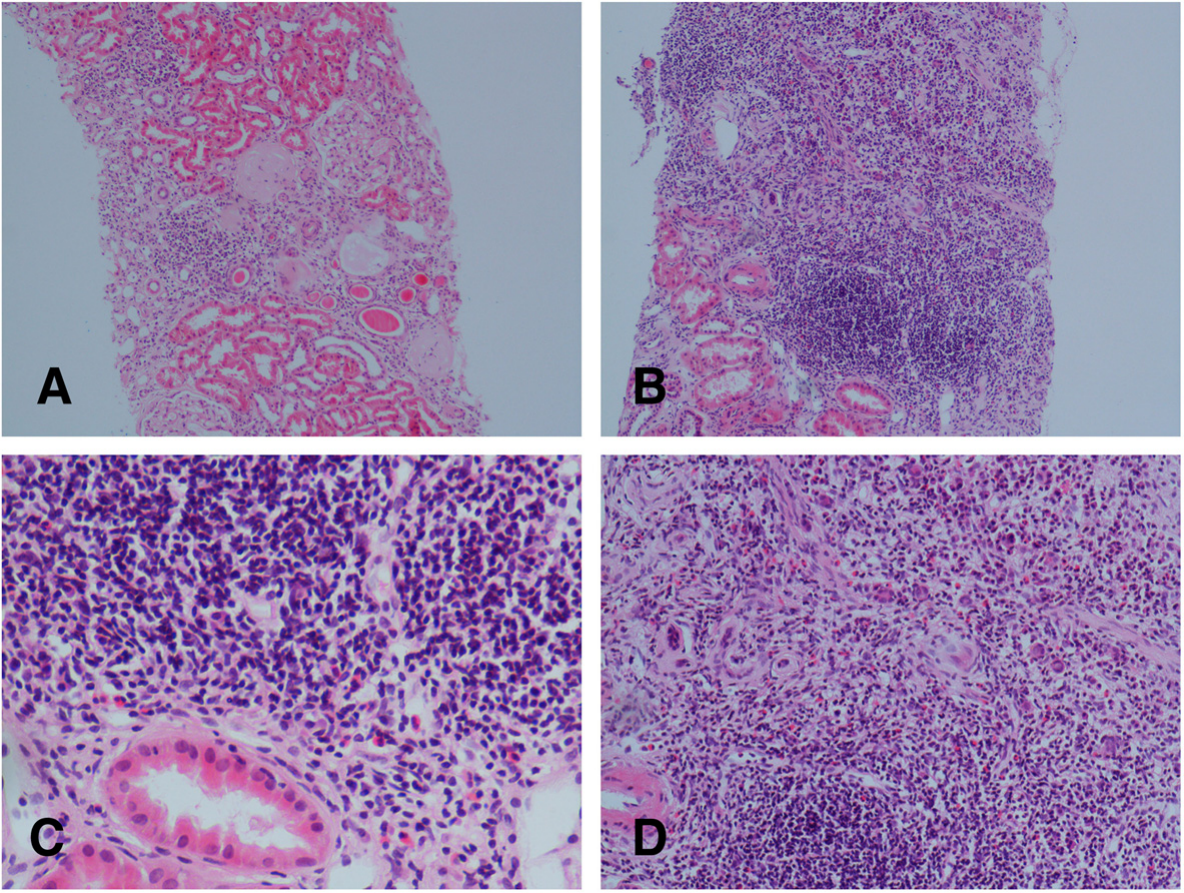
These panels are of representative hematoxylin and eosin stained slides from 2 patients showing the typical mononuclear lymphocytic inflammatory infiltrate of pSS TIN. Panels **a** & **b** are x10 magnification, panel **c** is x20 and panel **d** is x40 magnification

Thus, the infiltrate tended to be patchy, light to moderate in intensity and to affect the majority of the interstitium. Interstitial scarring was judged to be light to moderate (Fig. 2 for representative histology).

Three patients also had coexisting glomerular disease; one was coincidental thin basement membrane disease found on electron microscopy, the remaining two had mild glomerular mesangial matrix expansion.

**Fig. 3:**
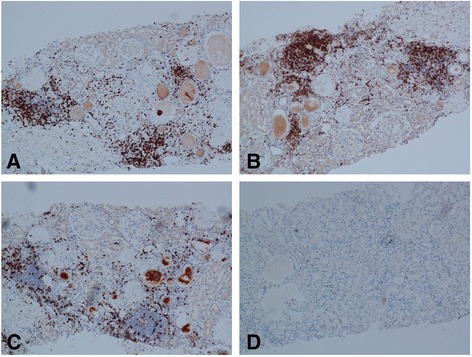
Shows representative pictures of slides immunostained for lymphocyte markers from the same patient. Panel **a** is stained for the T-cell marker CD3, panel **b** is stained for CD4, panel **c** is stained for CD8, and panel **d** is stained for the B-cell marker CD20

Immunophenotyping revealed that the inflammatory interstitial infiltrate was a T-cell infiltrate, and was predominantly CD4+, with lesser amounts of CD8+ cells and almost no CD20+ B-cells (see Fig. 3 for representative pictures).

Of the 12 patients, 11 were treated with MMF (median dose 1000 mg/day, 1000–1500 mg), 2 were treated with azathioprine (median dose 62.5 mg/day) and 1 had no immunosuppressive treatment at all. 9 patients were treated with Prednisolone (median starting dose 10 mg/day, 5–20 mg) weaned over 3–6 months. 4 patients remain on low dose Prednisolone (median dose 7.5 mg/day, 5–10 mg) in conjunction with MMF. The median duration of immunosuppressive treatment was 24 months (IQR 24).

**Fig. 4:**
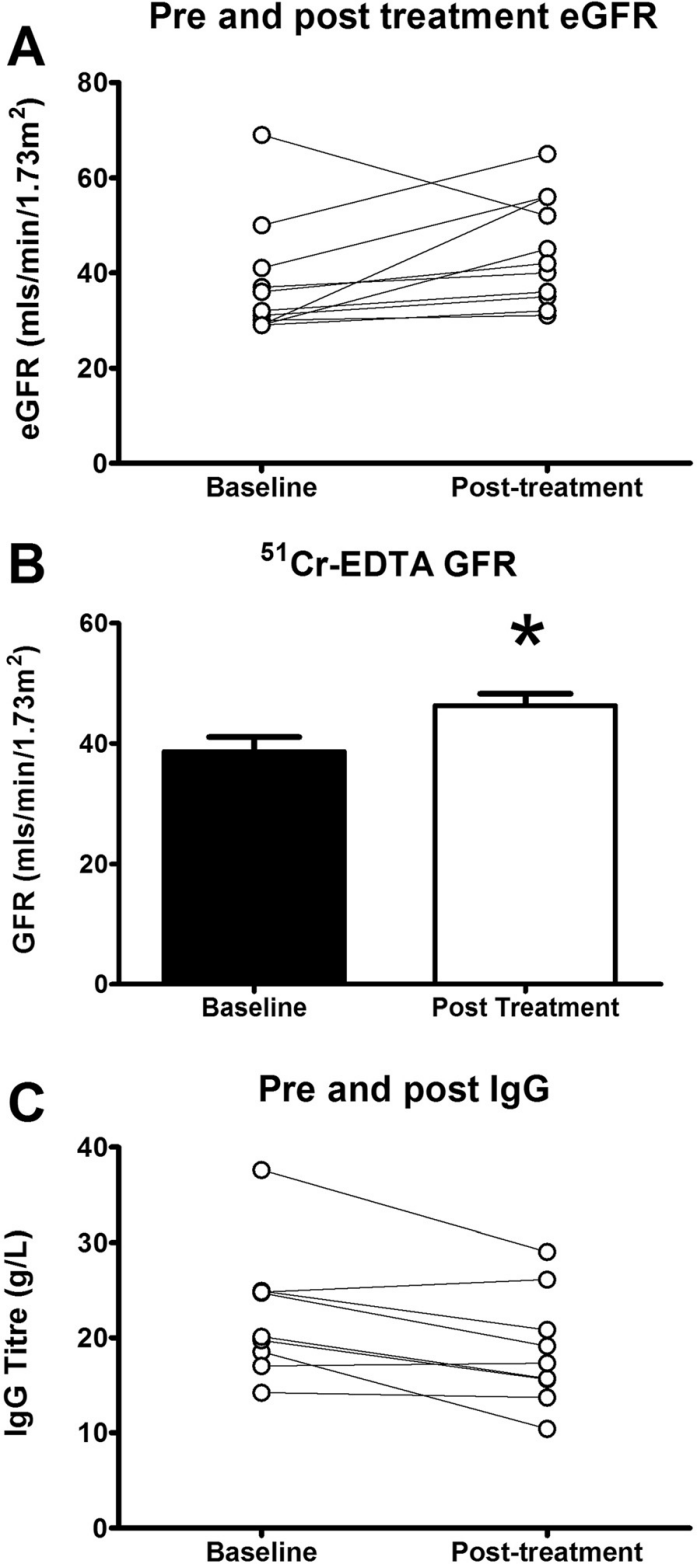
Shows data from treated patients. Panel **a** shows the MDRD eGFR pre and post treatment, panel **b** shows the measured ^51^Cr-EDTA GFR pre and post treatment in 6 of the patients, and panel **c** shows serum IgG levels pre and post treatment

Both serum creatinine and eGFR improved following treatment; the median serum creatinine decreased significantly following immunosuppression treatment (153 μmol/L, IQR 25, to 124 μmol/L, IQR 39, *p* = 0.02), as did the eGFR (32mls/min/1.73 m^2^, IQR 12 to 42mls/min/1.73 m^2^, IQR 21, *p* = 0.04). The GFR was directly measured in 6 patients, which also improved following treatment (37mls/min/1.73 m^2^, IQR 10.5, to 46mls/min/1.73 m^2^, IQR 6.5, *p* = 0.04). Serum IgG levels decreased significantly following treatment (20.1 g/L, IQR 7.1 to 17.3 g/L, IQR 8.8, *p* = 0.01) (Fig. 4).

## Discussion

Primary Sjögren syndrome has been estimated to affect 0.6 % of the US population (1.3 million US adults) [10] and has a worldwide distribution with consistent clinical characteristics [11].

Data on the prevalence of renal disease in pSS comes from two sources; retrospective studies looking for overt renal dysfunction, mainly defects in excretory renal function and proteinuria [5, 12, 13], and smaller prospective series in which tubular defects were explicitly screened for [3, 14–16]. While the retrospective studies found a rate of renal involvement in pSS of 4.3–6.5 % [5, 12, 13], the prospective studies observed a much higher rate of 28–42 % [3, 15].

The rarer glomerular lesions, typically the characteristic membranoproliferative glomerulonephritis (MPGN), occur due to immune complex deposition following B-cell expansion. These immune complexes are often cryoglobulins [5], which bind to vascular endothelium and may cause a small vessel vasculitis. This clinical picture occurs with clonal B-cell expansion, is usually associated with lymphoma, and tends to occur late in the course of the disease [4, 5].

The more typical tubulointerstitial nephritis can manifest in a number of ways, including with a mild to moderate reduction in the glomerular filtration rate, although progression to end stage renal failure with pSS TIN is reported [5]. The tubular dysfunction can manifest as proximal tubular dysfunction with low molecular weight proteinuria [3] or a full-blown renal Fanconi syndrome [17] (with bicarbonaturia, uricosuria, phosphaturia, glycosuria and low molecular weight proteinuria). Distal tubular disease may cause the characteristic distal renal tubular acidosis [18] (with hypokalaemia, nephrocalcinosis/kidney stones, osteomalacia and a variable metabolic acidosis) or nephrogenic diabetes insipidus [19] (NDI).

These tubular lesions often require special investigations to diagnose; assays for low molecular weight proteinuria (e.g. retinol binding protein), urinary acidification tests to diagnose dRTA and water deprivation tests to diagnose NDI may not be available outside of specialist centres.

We would advocate renal biopsy in all patients with pSS and tubular defects to confirm the diagnosis of TIN, and to distinguish from other potential causes of localised tubular defects (eg. the presence of light chain).

The TIN of pSS is characterized by an invasion of mononuclear lymphocytes. We found that these cells are predominantly CD4+ T-cells with lesser populations of CD8+ T-cells; this is in keeping with data from mouse models of renal pSS [20] as well as data in human renal tissue [21], although one study suggested that CD8+ T-cells are more dominant [22].

This pattern of epithelial inflammation is strikingly similar to that seen in labial salivary glands (LSGs), which are much more intensively studied. There is evidence that the infiltrating CD4+ T-cells may be pathogenic Th17 cells; IL-17 levels are elevated in the salivary glands and serum of pSS patients and these levels correspond to the severity of the histological lesion [23]. Furthermore, knocking out IL-17 prevents disease in a mouse model of Sjögren syndrome [24]. In more extensive histological disease in LSGs, B-cells become more pronounced in the infiltrate, and it has been suggested that treatment could be tailored to the type of infiltrating cell [25].

However, there are no proven effective systemic immunosuppressive treatments for pSS; the few randomized trials have been inconclusive or contradictory.

Typical treatment for patients with joint or skin disease with pSS, is often based on hydroxychloroquine or methotrexate. Resistant or extraglandular disease has been treated variously with corticosteroids, antiproliferative agents, calcineurin inhibitors, cyclophosphamide or B-cell depletion therapy [26].

The patients in this series were all treated with MMF; the rationale being that MMF would affect both T-cell and B-cell populations and should therefore be effective across the spectrum of pSS TIN. Indeed, MMF has been used successfully with acute TIN in other settings [27]. The optimal dose of MMF to use is unknown and the dose of MMF used in our series was relatively low. We adopted a pragmatic approach and escalated the MMF in the presence of persistent symptoms, or failure of recovery of renal function. We aimed where possible to normalise the serum IgG, as there is a well-recognised association between hypergammaglobulinaemic states and dRTA (originally reported by Morris and Fudenberg [28]) and TIN, even in the context of idiopathic hypergammaglobulinaemia [29]. We also used a short weaning course of corticosteroids, if there was no contraindication to do so, as suggested by two previous series [17, 30]. This regime was well tolerated, with only one patient unable to continue with MMF due to reversible leucopenia.

We have demonstrated a significant improvement in the MDRD eGFR in the patients treated in this way; this is the first series to do so in pSS TIN alone. That the measured ^51^Cr-GFR in 6 of these patients significantly improved suggests that this effect is real. Two other series have shown variable improvement in excretory renal function using corticosteroids and various immunosuppressants in a cohort that included both TIN and MPGN patients [17, 30]. We suspect that specific tubular defects occur relatively early within the disease process and during the stage of active inflammation. We aim to abrogate this inflammation prior to the development of significant fibrosis, and progressive renal disease.

### Limitations

This is a retrospective description of a relatively small number of patients from a single centre, and as such, there is no control population, and the investigations and treatment regimes are not completely uniform. As it is a description of pSS patients with TIN, the results are not necessarily generalisable to all patients with pSS.

## Conclusion

Patients with pSS TIN present with significant renal impairment and frequent tubular defects. The lesion is characterized by a mononuclear lymphocytic infiltrate that is predominantly composed of CD4+ T-cells and subsequent scarring. Treatment with MMF with or without a course of oral corticosteroids improved excretory renal function. Further studies into the pathogenesis and treatment of pSS TIN are warranted, given the frequency of this disease. Furthermore such studies may bring valuable insights into other inflammatory processes in the renal interstitium, such as acute allergic TIN and renal allograft rejection.

## Abbreviations

ANA: Antinuclear antibody
dRTA: Distal renal tubular acidosis
GFR: Glomerular Filtration Rate
eGFR: estimated Glomerular Filtration Rate
Cr-GFR: Chromium-51 labeled ethylenediamine tetraacetic acid measured GFR
IgG: immunoglobulin G
IQR: Interquartile range
LSGs: Labial salivary glands
LMW Proteinuria: Low molecular weight Proteinuria
MDRD eGFR: Modification of diet in renal disease equation for eGFR
MMF: Mycophenolate mofetil
MPGN: Membranoproliferative glomerulonephritis
NDI: Nephrogenic diabetes insipidus
pSS: Primary Sjögren syndrome
RBP: Retinol binding protein
TIN: Tubulointerstitial Nephritis
UCL: University College London
Urine PCR: Urine Protein:Creatinine Ratio
